# Relationship between Working Hours and Power of Attention, Memory, Fatigue, Depression and Self-Efficacy One Year after Diagnosis of Clinically Isolated Syndrome and Relapsing Remitting Multiple Sclerosis

**DOI:** 10.1371/journal.pone.0096444

**Published:** 2014-05-01

**Authors:** Peter Joseph Jongen, Keith Wesnes, Björn van Geel, Paul Pop, Evert Sanders, Hans Schrijver, Leo H. Visser, H. Jacobus Gilhuis, Ludovicus G. Sinnige, Augustina M. Brands, and the COGNISEC study group

**Affiliations:** 1 MS4 Research Institute, Nijmegen, The Netherlands; 2 Bracket, Goring-on-Thames, United Kingdom; Swinburne University, Melbourne, Australia; 3 Medisch Centrum Alkmaar, Alkmaar, The Netherlands; 4 Viecuri Medisch Centrum, Venlo-Venray, The Netherlands; 5 Amphia Ziekenhuis, Breda, The Netherlands; 6 Westfries Gasthuis, Hoorn, The Netherlands; 7 St. Elisabeth Ziekenhuis, Tilburg, The Netherlands; 8 Reinier de Graaf Gasthuis, Delft, The Netherlands; 9 Medisch Centrum Leeuwarden, Leeuwarden, The Netherlands; 10 Department of Experimental Psychology, Utrecht University, The Netherlands; Zuwe Hofpoort Ziekenhuis/Regionaal Psychiatrisch Centrum Woerden, The Netherlands; University Of São Paulo, Brazil

## Abstract

The role of cognitive domain dysfunction with respect to vocational changes in persons with Clinically Isolated Syndrome (CIS) and early Relapsing Remitting Multiple Sclerosis (eRRMS) is insufficiently known. We investigated thirty-three patients - 14 CIS, 19 eRRMS -, mean (standard deviation [SD]) time since diagnosis 13.5 (4.8) months and mean (SD) Expanded Disability Status Scale (EDSS) score 1.3 (1.1). Patients were assessed on the CDR System, a set of automated tests of cognitive function, which yielded scores for Power of Attention (ms), Continuity of Attention (#), Working Memory (SI), Episodic Memory (#) and Speed of Memory (ms). Work-related items and the confounding variables fatigue, depression, disease impact and self-efficacy, were assessed by self-report questionnaires. Patients had poorer Power of Attention compared to normative data (1187 [161.5] vs. 1070 [98.6]; P<0.0001) and slower Speed of Memory (4043 [830.6]) vs. 2937 [586.1]; P<0.0001). Power of Attention (Pearson r = −0.42; P<0.04), Working Memory (r = 0.42; P<0.04) and depression r = −0.41; P<0.05) correlated with number of days worked per week. Fatigue (r = −0.56; P<0.005), self-efficacy (r = 0.56; P<0.005) and disease impact (r = −0.46; P<0.05) correlated with number of hours worked per week. Persons who wished to work less had poorer Power of Attention (1247 vs. 1116 ms; P<0.02), those who wished to change job had poorer Episodic Memory (1.35 vs. 1.57; p<0.03). People who reduced working hours within 12 months after diagnosis had higher fatigue and disease impact, and lower self-efficacy. The findings of this pilot study indicate that one year after the diagnosis of CIS and RRMS Power of Attention and Speed of Memory are reduced, that Power of Attention and Memory are associated with a capability of working less hours, and that fatigue, depression and disease impact may negatively, and self-efficacy positively affect working hours.

## Introduction

Cognitive impairment is a disabling symptom in multiple sclerosis (MS), occurring in 45–65% of the patients [1]. It involves complex attention, information processing speed, (episodic) memory, and executive functions [1] [2]. In the clinically isolated syndrome (CIS) cognitive disturbances occur in 20–24% [3] of the patients in a pattern similar to that in relapsing remitting (RR) MS [4]. In early RRMS (eRRMS) the incidence of cognitive symptoms is 30% [5], and 4 years after diagnosis one out of three RRMS patients shows cognitive decline [6].

MS is furthermore associated with reduced working hours and high rates of unemployment [7]. About 4 years after diagnosis 30% of the patients are on disablement pension and 34% receive social services in relation to work [6]. Total disability, specific problems in mobility and hand function, fatigue, and cognitive symptoms all confer a risk of a change in vocational status [7]. The crucial role of cognitive impairment with respect to work is shown by the fact that worsening of cognitive symptoms [7] [8] and a decline on neuropsychological testing over time are predictive of a deterioration in employment status [9].

In recent years the Great Recession has worsened the socio-economic situation and public health in various countries [10]. The consequences, including lower income, decreased working hours and unemployment, may differ between countries and between population groups. Due to their disabilities and uncertain prognosis, persons with CIS and MS seem extra vulnerable to unwanted changes in vocational status. Up-to-date data on the impact of cognitive impairment on work in persons with CIS and eRRMS could enable preventive measures to be taken. Moreover, knowledge on the impact of cognitive domain dysfunctions on vocation may provide starting points for focused cognitive rehabilitation. Prevention of unemployment is beneficial to patients, in terms of income and health-related quality of life (HRQoL), and in terms of health-economics to the society as a whole.

In the ongoing Cognition and Socio-economics (COGNISEC) study we prospectively assess the relationship between cognition and socio-economics in patients with CIS and eRRMS, as well as the predictive value of early cognitive impairment with respect to socio-economic changes. To explore the relationship between cognitive domain dysfunction and working hours we analyzed the COGNISEC baseline data. We evaluated which domains of cognitive dysfunction are most associated with the number of hours worked per week, days worked per week, and maximum hours worked per day; we also investigated which domains are associated with a reduction of working hours or a job change after diagnosis, or a current wish to work less hours or to change job. Given the potential negative impact of other MS symptoms on vocation we also assessed fatigue, depression, disease impact and disability. Self-efficacy and HRQoL were additionally measured because the former may have a positive effect on work-related variables and the latter reflects an individual’s overall subjective well-being. The results of this pilot study are presented.

## Materials and Methods

### Study Design and Procedures

The COGNISEC study is a two-year, investigator-initiated, prospective, observational, multi-centre study being conducted in the Netherlands. The aims are to establish in individuals with diagnosis of CIS and eRRMS a) the relationship between cognition and socio-economics, and b) the predictive value of early cognitive worsening with respect to socio-economic changes. The study protocol was submitted to the Independent Review Board (IRB), an Ethics Committee (EC) in Amsterdam, the Netherlands. The IRB concluded that because of the observational design of the study a formal review by an EC was not required; the study not meeting the criteria stated in the Dutch Medical Research Involving Human Subjects Act of 1999 [11]. The study is being carried out in compliance with the Declaration of Helsinki.

Patients were recruited in out-patient departments of seven general hospitals. Patients who agreed to participate signed an informed consent form. The inclusion criteria were 1) diagnosis of CIS or RRMS according to the revised McDonald criteria [12], 2) maximum time since diagnosis two years, 3) maximum duration of disease modifying drug (DMD) treatment, if any, six months, 4) no relapse (i.e. no worsening of existing symptoms or occurrence of new symptoms), 5) clinically stable for at least 30 days, 6) written informed consent. The exclusion criteria were 1) worsening of symptoms suggestive of a relapse, 2) DMD treatment longer than six months, 3) progressive MS. The first patient was included on the 16^th^ of February 2010 and the last patient on the 5^th^ of January 2012. Primary outcomes of the present analyses are the correlations between cognitive domain scores and working hours.

### Cognitive Assessment

Cognition was assessed by the CDR System, a brief, multiple repeatable, computerized battery of cognitive tests, that has been validated in various disease states and cognitive disorders including dementia, epilepsy, sleep disorders and RRMS [13]–[15]. The battery uses alternate forms of tests and randomizes these across repeated assessments. The forms are conceptually equivalent and the use of randomization prevents systematic bias in comparison between visits when comparing between or within groups. To minimize the motor requirement in responding, patient responses are recorded via a simple response box with two large buttons, one marked ‘YES’ and one marked ‘NO’ in the patient’s own language. The patient is not required to use the computer keyboard or mouse and in the word recall tests oral responses are recorded by the test administrator [15]. The tests were administered by the MS nurses of the participating sites.

The CDR System is modular, and the selected battery measured attention and information processing speed (simple reaction time, choice reaction time and digit vigilance tasks – both accuracy of responding and reaction times), articulatory and visuo-spatial working memory (numeric and spatial working memory tasks) and verbal and visual episodic memory (immediate and delayed word recall, word recognition and picture recognition tasks) [15]. It took around 15–20 minutes to complete the tests.

Five domain scores were derived from the tests: Power of Attention, a measure of focused attention and information processing speed; based on the summed reaction times from the simple reaction time, choice reaction time and digit vigilance tasks (ms); Continuity of Attention, a measure of sustained attention, combining accuracy and error measures from the choice reaction time and digit vigilance tasks (#); Working Memory, summing accuracy measures from the numeric and spatial working memory tasks (sensitivity index [SI]); Episodic Memory, summing accuracy measures from the immediate and delayed word recall, word recognition and picture recognition tasks (#); and Speed of Memory, a measure of complex information processing speed, summing reaction times from the two working memory and the two episodic recognition tasks (ms) [15] [16] (CDR domains and tasks S1). Power of Attention and Speed of Memory are measured by response latencies and smaller scores indicate better function. Continuity of Attention, Working Memory, and Episodic Memory are accuracy scores and higher scores indicate better function. Normative age-matched (range 20–52 years) data from healthy volunteers were taken from the CDR System database and were used as control. These volunteers had participated in early stage clinical trials to establish the safety of new medicines in healthy normal individuals and were free of any medical or physical illness and not receiving any medications which might affect cognitive function. A validation study of this CDR battery in RRMS patients demonstrated the test-retest reliability over repeated assessments, and significant correlations between domain scores and the Digit Symbol Substitution Test, the Paced Auditory Serial Addition Test, the Multiple Sclerosis Functional Composite, and the Expanded Disability Status Scale (EDSS) [15].

### Assessment of Work-related Variables

In the COGNISEC study socio-economic variables are assessed by use of the Multiple Sclerosis Socio-Economic Questionnaire (MSSEQ). The MSSEQ is a self-report tool based on the ‘Beeld van de Nederlandse Bevolking (Picture of the Dutch Population) 2009’ questionnaire of the Department of Sociology, Radboud University Nijmegen, the Netherlands [17], and consists of 5 modules: Household and Finances (module A; 12 items), Children (module B; 21 items), Education (module C; 17 items), Work (module D; 75 items) and Relations and Leisure (module E; 21 items). Items are rated binary (yes or no), numerically, categorically, or reported as text in open fields. Eight items in module D relate to the present study and were included in the analyses (Table 1). The questions regarded the work situation at the time of diagnosis (question A1), the first 12 months after diagnosis (questions A2–A3), the current situation (questions B1–B3) and wishes for the immediate future (questions B4–B5).

**Table 1:** Work-related questions.

A. If you had a job at the time of diagnosis, either as employee or as self-employed person
1. How many hours per week did you work?2. Did you work less hours per week within 12 months after diagnosis due to CIS or MS?3. Did you change your job within 12 months after diagnosis due to CIS or RRMS?
**B. If you work at present, either as employee or self employed person**
1. How many hours per week do you work?2. How many days per week do you work?3. What is the maximum number of hours you work per day?4. Do you wish to work less hours per week due to CIS or RRMS?5. Do you wish to change your job due to CIS or RRMS?

### Assessment of Confounding Variables and HRQoL

Fatigue, depression, self-efficacy, impact of disease, and disability are aspects of MS that have been related to changes in vocation [18]. HRQoL is a subjective measure of total wellbeing and may be negatively affected by changes in employment in persons with MS.

*Fatigue.* Fatigue was assessed by the Modified Fatigue Impact Scale-5 Item Version (MFIS-5). The MFIS-5 is part of the Multiple Sclerosis Quality of Life Inventory, developed by the Consortium of Multiple Sclerosis Centers Health Services Research Subcommittee [19]. The MFIS-5 is a validated questionnaire examining a patient’s perception of fatigue over the past month. It is derived from the Fatigue Impact Scale, a 40-item multidimensional scale, that assesses the perceived impact of fatigue on a variety of daily activities [20]. For both scales answers to each question are rated on a 5-point scale from 0 to 4. The MFIS-5 total score consists of the sum of the raw scores and ranges from 0 to 20, where higher scores indicate more experienced fatigue [19].

#### Depression

Depression was measured with the Beck Depression Inventory (BDI). The BDI is a short validated questionnaire of 13 questions relating to depressed mood [21] [22]. Answers are rated on a 4-point scale from 0 to 3. Total scores range from 0 to 39 and higher scores indicate more depressed mood.

#### Self-efficacy

Self-efficacy was assessed with the Multiple Sclerosis Self-Efficacy Scale (MSSES) [23]. The MSSES is a validated scale for the measurement of self-efficacy in MS and consists of two subscales of 9 questions each [23]. Answers to each question are rated on a 10-point scale from 10 to 100. Each subscale score is the mean of the 9 item scores and the MSSES score is the summation of both subscale scores, ranging from 20 to 200.

#### Impact of disease

The impact of disease was assessed by the Multiple Sclerosis Impact Scale-29 (MSIS-29) [24]. The MSIS-29 is a psychometrically validated patient-based rating scale measuring the physical and psychological impact of MS from the patient’s perspective [24], [25]. It is considered a valuable outcome measure in intervention studies of patients with MS [26]. Each item is scored on a 5-point scale from 1 to 5. The addition of all item scores yields the MSIS-29 score, ranging from 29 to 145.

#### Disability

Disability was measured with the EDSS [27], a measure widely used in MS clinical studies [28]. The EDSS is a single, ordinal, non-linear composite score based on observer-rated scales in seven neurologic functional systems, and ambulation [28]. The EDSS scores range from 0 to 10 with steps of 0.5 and a higher score indicates more disability.

#### HRQoL

HRQoL was measured by the Leeds Multiple Sclerosis Quality of Life (LMQoL) questionnaire. The LMSQoL is a validated self-assessment scale that consists of eight questions, examining MS-related aspects of QoL over the past month [29] [30]. Answers are rated on a 5-point scale from 1 to 4. The resulting score ranges from 8 to 32, with higher scores reflecting higher levels of wellbeing [29] [30].

### Statistics

Pearson correlation coefficients were calculated between Power of Attention, Continuity of Attention, Working Memory, Episodic Memory, and Speed of Memory scores and work-related variables and also between MFIS-5, BDI, MSSES, MSIS-29, EDSS, and LMSQoL scores and work-related variables. To explore to which extent the variance in working hours is explained by cognition, fatigue, depression, self-efficacy, and disease impact we performed a stepwise regression analysis for work-related variables with one or more cognitive scores. We adopted the standard alpha for variables to enter the regression model of (P<0.15). The order of entering the variables was determined by the strength of the correlation with the outcome variable. For comparisons between groups Student’s *t*-test were applied. Statistical tests were performed using the SPSS Statistics 17.0 package. P values <0.05 were considered significant. Given the explorative character of the study a correction for multiple testing has not been performed [31], [32].

## Results

### Patient and Disease Characteristics

Fifty persons were included in the COGNISEC study. Three had to be excluded from the data set as the time since diagnosis exceeded two years (n = 2) or the disease course was progressive (n = 1). Forty of the 47 persons in the data set provided information on their vocational status. Seven (17.5%) of these reported having no occupation: three were student, two were on sick leave, one had no job by choice, and one received disablement benefit. Thirty-three (82.5%) persons had a job and constituted the study group; 25 were female and 8 male. The mean (standard deviation [SD]) age was 39.8 (8.5) yrs., and mean (SD) time since diagnosis 13.5 (4.8) months. Fourteen persons had CIS and 19 eRRMS. Eight persons were treated with a DMD: six with sc. interferon-beta-1a and two with sc. interferon-beta-1b.

The education levels, according to the highest diploma obtained, were classified as low, middle, higher and academic. The respective levels were seen in 4, 13, 12 and 4 persons. The types of work in the low education group: administration personnel department, domestic help, assembly operator and supermarket worker; in the middle education group: law court worker, administration worker, sales representative (twice), nurse, receptionist, tutor mentally retarded persons, garage keeper, shopkeeper, metalworker, cheesemaking farmer, caring for persons with dementia, and home care; in the higher education group: personnel manager, maintenance technician, primary school teacher, nurse (twice), operator, shopkeeper, caring for mentally retarded persons, communication worker, management assistant, stockbroker, and secretary; in the academic group: bank employee, school manager, primary school teacher, and ICT project leader.

### Cognitive Performance

Mean (SD) scores of Power of Attention, Continuity of Attention, Working Memory, Episodic Memory, and Speed of Memory are presented in Table 2. Compared to the age-matched control population the Power of Attention and Speed of Memory scores were significantly poorer in the patient group (both P<0.0001), these deficits having Glass’ effect sizes of 1.2 and 1.9 respectively and thus having everyday relevance, both exceeding the criterion of a large effect size (0.8). Speed of Memory was worse (mean score 4343.4 [SEM 145.9]) in the CIS subgroup as compared to the eRRMS (mean 3839.4 [SEM 115.8]; P<0.01) subgroup.

**Table 2:** Mean (SD) cognitive domain scores in patients and in the normative data set.

	CIS and eRRMS (N = 33)	Normative data (N = 1409)	P	Glass’ Effect Size
Power of Attention (ms)	1187 (161.5)*	1070 (98.55)	0.0002	1.2
Continuity of Attention (#)	91.96 (3.162)	90.97 (3.096)	0.0818	0.3
Working Memory (SI)	1.844 (0.222)	1.790 (0.194)	0.1764	0.3
Episodic Memory (#)	1.505 (0.253)	1.343 (0.320)	0.001	0.5
Speed of Memory (ms)	4043 (830.6)*	2937 (586.1)	<0.0001	1.9

### Work-related Variables

At the time of diagnosis the mean (SD) number of hours worked per week was 29.95 (10.88) and at the time of the study 25.16 (11.40) (P>0.05). At the time of the study the number of days worked per week was 3.84 (1.22) and the maximum hours worked per day 7.28 (2.34). Four-teen (45%) of 31 persons reported to have switched to less hours per week within 12 months after diagnosis, whereas four (12%) of 32 persons changed their job within 12 months after diagnosis. Eight (31%) of 26 persons wished to work less hours and eight (32%) of 25 persons wished to change their job.

### Confounding Variables and HRQoL

Mean (SD) value of MFIS-5 scores was 8.64 (4.20), of BDI 4.32 (3.45), MSSES 162 (26.31), MSIS-29 53.67 (17.88), EDSS 1.31 (1.10), and LMSQoL 21.93 (3.39).

### Correlative Analyses

The correlation coefficients for the relationship between cognitive scores and working hours are presented in Table 3. The Power of Attention and Working Memory scores correlated significantly with the number of days worked per week (r = 0.42 and r = −0.42 respectively, both P = 0.04). The BDI score also correlated with the number of days worked per week (r = −0.41), whereas the MFIS-5 (r = −0.56), MSSES (r = 0.56), and MSIS-29 (r = −0.46) scores correlated with the number of hours worked per week (Table 4). In addition, the MSIS-29 score correlated with the maximum hours worked per day (−0.44). Moreover, the education level correlated with Working Memory (−0.41; p = 0.01) and with Episodic Memory (0.41; p = 0.02), and less so with Continuity of Attention (0.35; p = 0.04).

**Table 3:** Correlations between cognitive scores and working hours.

	Hours per week (n = 27)	Days per week (n = 25)	Max. hours per day (n = 26)
Power of Attention (ms)	−0.30	−0.42*	−0.04
Continuity of Attention (#)	−0.06	−0.09	0.18
Working Memory (SI)	0.30	0.42*	0.20
Episodic Memory (#)	0.22	0.29	0.30
Speed of Memory (ms)	−0.01	0.04	−0.01

Max., maximum; *, P<0.04; all other P values >0.125.

**Table 4:** Correlations between confounding variables and HRQoL, and working hours.

	Hours per week	Days per week	Max. hours per day
MFIS-5	−0.56* (n = 24)	−0.34 (n = 22)	−0.38 (n = 23)
BDI	−0.39 (n = 26)	−0.41** (n = 24)	−0.34 (n = 25)
MSSES	0.56* (n = 24)	0.14 (n = 22)	0.32 (n = 23)
MSIS-29	−0.46** (n = 24)	−0.19 (n = 22)	−0.44** (n = 23)
EDSS	0.12 (n = 21)	0.11 (n = 20)	0.17 (n = 20)
LMSQoL	0.28 (n = 23)	0.22 (n = 21)	0.21 (n = 22)

Max., maximum;

*P<0.005;

**P<0.05.

### Comparative Analyses

Persons (eight of 26) who wished to work less hours had a poorer Power of Attention (scores: 1247 vs. 1116 ms; P<0.02), and those (eight of 25) who wished to change job had a poorer Episodic Memory (scores: 1.57 vs. 1.35; p<0.03). Individuals (14 of 22) who switched to less working hours within 12 months after diagnosis had higher mean MFIS-5 (10.27 vs. 6.87; P<0.05) and MSIS-29 (62.2 vs. 45.6; P<0.02) scores, and a lower MSSES score (89.2 vs. 97.4; P<0.02) than those who did not.

### Regression Analyses

On stepwise regression analysis the MFIS-5, Episodic Memory, and Working Memory scores accounted for 52.6% of the variance in the maximum hours worked per day (partial R-Square values 0.271, 0.110, and 0.146 resp.). The MFIS-5 and the Episodic Memory scores accounted for 47.9% of the variance in the hours worked per week (partial R-Square values 0.370 and 0.110, resp.).

## Discussion

In persons with CIS and eRRMS we investigated whether working hours or vocational changes after diagnosis related to specific domains of cognitive dysfunction or measures of psychological wellbeing. Assessments at 13.5±4.8 (mean±SD) months after diagnosis showed, first, positive correlations between the number of days worked per week, and power of attention and working memory; second, persons who wished to work less hours had a worse power of attention and those who wished to change job had a worse episodic memory; third, negative correlations between working hours and fatigue, disease impact, and depression, and a positive correlation between working hours and self-efficacy; fourth, individuals who switched to less working hours within 12 months after diagnosis had higher fatigue and disease impact, and less self-efficacy.

Attention and memory have been reported to be impaired in persons with CIS and eRRMS [1]. Schulz et al. showed that, compared with healthy controls, persons with eRRMS show lengthened reaction times for simple and focused attention (19–38%) and impaired non-verbal memory function (33%) [33]. A recent study in CIS and MS (disease duration six years or less) found deteriorations on working memory [34]. Our correlative data suggest that attention and working memory may have a negative impact on working hours one year after the diagnosis CIS or RRMS. These findings were paralleled by the outcomes of the regression analyses, that identified working memory and episodic memory as major determinants. Actually, vocational activities require both the short-term use of memory and attention - to complete tasks or execute challenges relating to executive functions -, and the retrieval of contextual information pertaining to specific events or experiences. However, at the group level power of attention and speed of memory were poorer compared to normative data, whereas working memory and episodic memory were not impaired. It could be the case that in a vocational setting unimpaired working memory has to compensate for other symptoms, like fatigue, and thus becomes the limiting factor of working hours. On the other hand, it has been shown that MS patients may achieve a normal performance on working memory tasks at the cost of more prefrontal network activity [35]. Consequently, normal test results may overestimate memory functions, and as soon as situations become more strenuous undetected impairments may manifest. It could therefore be conceived that in demanding vocational situations a working memory dysfunction that went unnoticed on neuropsychological testing does become evident and clinically relevant.

The variance in hours worked per week and in maximum hours worked per day was partly explained by fatigue. In fact, working hours correlated more strongly with fatigue, self-efficacy, and disease impact than with cognitive scores. Moreover, people who reduced their working hours within 12 months after diagnosis had higher current levels of fatigue and lower levels of self-efficacy than those who had not done so. Fatigue is a chronic symptom of MS, often present from disease onset, whereas self-efficacy is an individual trait. Given the short interval between diagnosis and study assessments we expect these variables not to have changed significantly. Therefore, the retrospective data suggest that fatigue and low self-efficacy played a role in the decision to reduce the working hours. In a study by Smith and Arnett 90% of patients working reduced hours reported that fatigue was a primary symptom responsible for their work status change, whereas in the non-working group 86% reported that broad physical-neurological symptoms were responsible for their change in work status [36]. A recent study showed that a low level of fatigue is a significant predictor of work capacity [37]. So, to prevent persons with CIS and eRRMS from having to reduce their working hours in the first year after diagnosis an optimal treatment of fatigue, as well as an heightened self-efficacy seem helpful. Likewise, the correlations between working hours, and depression and disease impact suggest that treatment of depressive symptoms and early prevention of disease activity by DMDs may increase patients’ chances to preserve their working hours. It is of note that, according to a recent report, in MS patients with minor disability (EDSS< = 3.0) depressive symptoms indeed seem to have a major impact on employment status [38].

The observation that the EDSS score did not correlate to working hours is understandable. In the lower range of the EDSS – in our patients the mean (SD) score was 1.3 (1.1) – the score is mainly based on physical-neurological signs, and as mentioned above, working reduced hours is not primarily caused by such abnormalities [36]. In addition, the factors that related to working hours (cognition, fatigue, depression) are insufficiently measured by the EDSS.

In spite of the correlations we found, about half of the variance in working hours (per week, maximum per day) was not explained by our data. The following factors might also have been operative: specific job characteristics [39], level of education [36], [39], occupational attainment [40], occupational prestige [36], [39], company policy, and interpersonal relationships between patients and colleagues and superiors. With respect to the latter we mention that the capability to reason about the mental state of others (theory of mind) and the ability to have insight into emotional stages and feelings of others (empathy) may be deficient at the early stages of RRMS [41], [42] even in patients who have no substantial neuropsychological deficits [42]. These social cognitive impairments may cause interpersonal problems [42]. Emotional prosody comprehension may also be deficient in eRRMS [43], which makes misunderstandings and poor communication, and consequently strained interpersonal relationships, even more likely [43].

Our study has several limitations. The number of patients was rather low and the study’s external validity is limited, as all patients were seen in general hospitals in the Netherlands. So, international studies in larger patient groups, recruited in general and academic centers, are needed to corroborate our findings. Furthermore, one might question the suitability of self-report outcomes in MS patients with cognitive dysfunction [44]. Importantly, memory dysfunction has been shown not to compromise the reliability or validity of self-reported questionnaires [44]. Moreover, working memory and episodic memory were not impaired in our patients. By using short questionnaires we tried to minimize the risk that cognitive dysfunction, like impaired power of attention, would interfere with the completion of the questionnaires.

In all, our data suggest that within one year after diagnosis persons with CIS or RRMS have experienced, or do express a wish for, vocational changes and that these changes are related to their disease, especially cognition, fatigue, depression and disease impact. For everyday clinical practice our findings imply that, to prevent in persons with CIS or eRRMS unwanted vocational changes, several measures are to be taken: first, close monitoring of cognitive function, especially attention and memory, starting after diagnosis [45]; second, focused cognitive rehabilitation in persons with low or impaired attention or memory functions; third, assessment and adequate treatment of fatigue and depression; fourth, considering an early start of DMD treatment to prevent the occurrence or further increase of disabilities and disease impact [46]; and, fifth, considering interventions that may increase the self-efficacy with respect to management of symptoms. Recently, Hankomaki et al. performed a longitudinal study in 36 newly diagnosed MS patients and found after 6.1 (mean) years follow-up a significant decline in divided attention (dual task) and information processing speed, but no significant deterioration in overall cognitive performance [47]. The authors conclude that, given the impact of cognitive impairment on patients' quality of life, early detection of its occurrence in MS is extremely important [47].

To be optimally effective, the set of measures we suggest should preferably be implemented as preventive health care programs. However, as a result of the Great Recession, political decision makers in many countries have taken austerity measures that substantially curtail preventive programs with demonstrably negative effects on health status [48]. It is therefore to be expected that only the pressure of patient organisations and health care professionals will lead to structural measures that may prevent the occurrence of vocational changes in persons with CIS ore RRMS [49]. Studies that on a larger scale confirm our findings would be of help.

## Conclusion

Our data indicate that one year after the diagnosis of CIS and RRMS power of attention and speed of memory are reduced, that power of attention and memory are associated with a capability of working less hours, and that fatigue, depression and disease impact may negatively, and self-efficacy positively affect working hours.

## Supporting Information

Table S1 (DOC)
