# Correction: Relationship between Working Hours and Power of Attention, Memory, Fatigue, Depression and Self-Efficacy One Year after Diagnosis of Clinically Isolated Syndrome and Relapsing Remitting Multiple Sclerosis

**DOI:** 10.1371/journal.pone.0105654

**Published:** 2014-08-06

**Authors:** The PLOS ONE Staff

There is an error in the author group affiliation. The correct sentence is: Membership of the COGNISEC Study Group is provided in the Acknowledgments.
