# Supplementary material for: Relationship between Working Hours and Power of Attention, Memory, Fatigue, Depression and Self-Efficacy One Year after Diagnosis of Clinically Isolated Syndrome and Relapsing Remitting Multiple Sclerosis
Source: PLoS One. 2014 May 1;9(5):e96444. doi: 10.1371/journal.pone.0096444 (PMC4006840; doi:10.1371/journal.pone.0096444)
Supplement: Table S1 — (DOC) [file pone.0096444.s001.doc]

**Table S1.** CDR cognitive domains (units), tasks and derivation of scores.

| **Power of Attention (ms)**  A measure of attention and psychomotor/information processing speed summing reaction times from the *Simple reaction time, Choice reaction time* and *Digit vigilance tasks*. Derivation: Simple Reaction Time + Digit Vigilance Speed + Choice Reaction Time.  **Continuity of Attention (#)**  Measure of attention summing accuracy and error measures from the *Choice reaction time* and *Digit Vigilance tasks.* Derivation: (Digit Vigilance Targets Detected *0.45) + (Choice Reaction Time Accuracy *0.5) – Digit Vigilance False Alarms.  **Working Memory (SI)**  Measure of working memory summing accuracy measures from the *Numeric* and *Spatial working memory tasks.* Derivation: Spatial Working Memory Sensitivity Index + Numeric Working Memory Sensitivity Index.  **Episodic Memory (#)**  A measure of long-term memory summing accuracy measures from the *Immediate* and *Delayed word recall, Word recognition* and *Picture recognition tasks*. Derivation: (Word Recognition Original Stimuli Accuracy + Word Recognition Novel Stimuli Accuracy - 100) + (Picture Recognition Original Stimuli Accuracy + Picture Recognition Novel Stimuli Accuracy - 100) + ((Immediate Word Recall Words Correctly Recalled - Immediate Word Recall Errors) * 100 / 15) + (( Delayed Word Recall Words Correctly Recalled - Delayed Word Recall Errors) * 100 / 15).  **Speed of Memory (ms)**  A measure of complex information processing speed summing reaction times from the *Numeric* and *Spatial working memory* and *Word and Picture recognition tasks.* Derivation: *Spatial Working Memory Speed + Numeric Working Memory Speed + Word Recognition Speed + Picture Recognition Speed.* |
| --- |
